# Concurrent and Construct Validity of the Diagnoform^®^ Kid Physical Fitness Test Battery in Primary School Children

**DOI:** 10.3390/jfmk11030256

**Published:** 2026-06-27

**Authors:** Alexis Barbry, Jérémy Coquart, Caroline Verhoeven, Malgorzata Klass

**Affiliations:** 1 Laboratoire Lorrain de Psychologie et Neurosciences de la Dynamique des Comportements, Université de Lorraine, F-57000 Metz, France; alexis.barbry@univ-lorraine.fr; 2 Unité de Recherche Pluridisciplinaire Sport Santé Société, University of Lille, University of Artois, University of the Littoral Opal Coast, F-59000 Lille, France; jeremy.coquart@univ-lille.fr; 3 Department of Mathematics Education, Faculty of Medicine, Université libre de Bruxelles, 1070 Brussels, Belgium; caroline.verhoeven@ulb.be; 4 Research Unit in Cardiorespiratory Physiology, Exercise and Nutrition, Faculty of Human Movement Sciences, Université libre de Bruxelles, 1070 Brussels, Belgium

**Keywords:** cardiorespiratory fitness, agility, muscular power, speed, child

## Abstract

**Background**: Physical Fitness (PF) is an important health marker that should be regularly monitored with simple, time-efficient test batteries such as Diagnoform^®^ Kid. However, its validity remains insufficiently evaluated. Therefore, this cross-sectional study examined its concurrent and construct validity against a battery of established reference tests. **Methods**: 184 children were grouped by age (6–7, 8–9 and 10–11 years). The Diagnoform^®^ and reference batteries assessed identical PF components using the following tests: 6-min shuttle run-walk vs. reduced Cooper for cardiorespiratory fitness, hopscotch vs. quadrant jump for agility, standing broad jump with vs. without execution instructions for power, 5-s sprint vs. 20-m sprint for speed, and simplified fingertip-to-floor vs. sit-and-reach for flexibility. Concurrent validity was examined using bivariate correlations, and construct validity through responsiveness to age and sex. **Results**: Corresponding tests were significantly correlated for all PF components (*p* < 0.001), except for agility at 10–11 years (*p* = 0.094). Agility showed moderate correlations in the younger groups (*r* = −0.45 to −0.67). Correlations were strong for cardiorespiratory fitness and flexibility (*r* ≥ 0.70). For power and speed, correlations ranged from moderate (power: *r* ≥ 0.61 at 6–7 and 8–9 years; speed: *r* = 0.58 at 6–7 years) to strong (power: *r* ≥ 0.83 at 10–11 years; speed: *r* ≥ 0.70 at 8–9 and 10–11 years). Age and sex influenced performances in both batteries, except for agility (no sex effect observed) and flexibility (sex effect observed in both batteries, whereas an age effect was found only for boys on the reference test). **Conclusions**: Despite slightly lower responsiveness of the simplified fingertip-to-floor test to age, findings support the concurrent and construct validity of the Diagnoform^®^ Kid tests.

## 1. Introduction

Physical Fitness (PF), a set of individual characteristics reflecting the ability to perform Physical Activity (PA) and/or to achieve sport performance, is widely recognized as a powerful health marker [1,2,3]. Therefore, the literature highly recommends to regularly assess PF level to monitor the health status of children [1,3]. To fulfill this public health mission, schools might play a crucial role by helping to identify children with a low PF for promoting healthy behaviors from the younger age [1,3,4]. However, primary school physical education teachers report a lack of time and resources [5]. Consequently, it is crucial to offer a PF assessment battery that is inexpensive, easy, and quick to administer, enabling professionals to efficiently evaluate children’s PF [2].

For measuring PF level, the American College of Sports Medicine (ACSM) recommends assessing both health-related (i.e., body composition, CardioRespiratory Fitness [CRF], muscular fitness, flexibility) and skill-related (e.g., agility, speed) PF components [2]. For measuring these PF components, professionals generally use field-based PF tests, which were specifically developed to facilitate PF assessment in school sports halls and other settings [2,6,7,8]. These field tests have several advantages: they are easy to administer to groups, require little time, and are low cost [2,6]. Nevertheless, considering children’s specific characteristics, some field tests present notable limitations [9]. For example, certain tests are not well aligned with children’s natural activity patterns (e.g., continuous exercises are not in line with the children’s natural tendency toward intermittent exercises) [9,10]. Moreover, several field tests are too time-consuming and require specialized equipment (e.g., audio pacing systems) [8]. These disadvantages may further complicate the implementation of PF assessments [8,9]. To facilitate their use, PF batteries should align with children’s natural activity patterns, require minimal equipment, and be as time-efficient as possible [2]. In addition, the ACSM also recommends that PF batteries provide age- and sex-related reference values (e.g., percentile values) to efficiently screen children’s PF level [2,11,12].

Several PF batteries for children have been developed by the scientific community [13]. Nevertheless, some PF tests included in these PF batteries require specific equipment, may not be aligned with children’s natural activity patterns, and the time required to complete these batteries may exceed what is feasible during regular physical education lessons [8,13]. More recently, the Diagnoform^®^ Kid (DIAG; IRFO, Loos, France; https://irfo.fr/programme-diagnoform/diagnoform-kid/; accessed on 31 May 2026) has recently gained increasing attention, because it closely aligns with the ACSM guidelines mentioned above and meets all the criteria described earlier [2,4,12,14,15,16,17,18]. Indeed, it includes both health- and skill-related PF components assessments [2,4,12], is easy to administer (e.g., by primary school teachers), time-efficient (i.e., ~60 min for 20 children), adapted to children’s natural activity patterns, and requires little equipment [2,4,12,18]. These advantages have enabled the DIAG to be used to assess approximately 70,000 French and Belgian children aged 6 to 11 up to 2026 [4,12]. This large amount of data made it possible to establish age- and sex-specific reference values [4,12]. Additionally, a dedicated digital platform enables to instantly generate a PF report for an individual child, or an entire class, and to compare results with peers of the same age and sex [4,12]. Finally, the five DIAG tests demonstrated moderate to good test-retest reliability in children (i.e., intraclass correlation coefficient ≥ 0.60) [18,19]. Despite these benefits, the validity of the PF tests included in the DIAG has not yet been verified. Validity (i.e., the ability of a test to measure the PF component it is supposed to assess) [20] is considered an important factor in making a field test an accurate method for measuring a PF component [2,21]. Two types of validity are generally assessed: concurrent validity (i.e., a strong correlation with a criterion measure or a recognized field test) and construct validity (i.e., the ability of a test to distinguish between groups presenting an expected difference in performance; e.g., younger vs. older children) [20,21,22].

The aim of this cross-sectional study was therefore to examine the concurrent and construct validity of the DIAG tests. We assumed that: (i) for each age group, performance achieved at each DIAG test would be correlated with the performance of the reference (REF) test, and (ii) both the DIAG and REF field tests would similarly detect the effects of age and sex.

## 2. Materials and Methods

### 2.1. Participants

For the recruitment, 4 public schools and a non-profit association organizing activities for children during school holidays, all located in Brussels (Belgium), were contacted between September 2023 and November 2024. These institutions were recruited using a convenience sampling approach for practical reasons (i.e., proximity to the university, availability of a sports hall, and pre-existing collaborative relationships). The inclusion and exclusion criteria are detailed in Table 1. Prior to the study, written informed consent was obtained from all children and their parents. This study was conducted in accordance with the principles of the Declaration of Helsinki and was approved by the Ethics Committee of the Erasme Hospital (approval code: P2023/357/B4062023000203; approval date: 27 November 2023).

To examine the effect of age, 3 age groups were defined: 6–7 years (mean age: 6.4 ± 0.5 years), 8–9 years (mean age: 8.3 ± 0.5 years), and 10–11 years (mean age: 10.8 ± 0.4 years) [23]. Based on the main aim of this cross-sectional study, a sample size calculation was performed using G*Power 3.1 (University of Düsseldorf, Düsseldorf, Germany) for correlation analysis (bivariate normal model) [24,25]. The calculation suggested a minimal sample size of 46 by age group is needed to be able to detect a correlation coefficient of 0.4 (i.e., moderate correlation, considered the minimum threshold for supporting concurrent validity between two physical fitness tests) [26,27] with two-tailed alpha error of 0.05 and power of 80%. All children who met the above inclusion criteria were included, allowing additional participants to compensate for potential dropouts.

### 2.2. Procedure

Each child completed the 2 PF test batteries (i.e., DIAG and a battery composed of REF tests) in a randomized order, with a 7-day interval between them. Each school and the association were randomly allocated, through a simple random draw conducted by the main investigator, to one of the two test-order sequences: (i) DIAG battery followed by the REF battery, or (ii) the REF battery followed by the DIAG battery.

To limit confounding factors and ensure inter-session reliability, the instructions of the international ACSM guidelines [2] were followed: (a) the PF tests of both batteries were performed in the exact same order according to the PF component measured (i.e., CRF, agility, muscular power, speed, and flexibility), (b) both PF batteries were performed in the same environment (i.e., sport halls), (c) the PF tests were supervised by 2 investigators (MK and AB), both holding PhDs in sports science and possessing extensive experience in PF assessment, with assistance from bachelor’s and master’s students in physical education, and (d) strong verbal encouragement was provided [2,28]. In line with the ACSM guidelines, age (year), body mass (kg), and height (cm) were also measured. Body Mass Index (BMI, kg·m^−2^) was then calculated (body mass in kg by height in m squared) because it is included in the DIAG battery as an anthropometric indicator of body composition (i.e., a health-related PF component) [2,4,29].

### 2.3. Diagnoform^®^ Kid

The DIAG is composed of 5 PF tests measuring different health- and skill-related PF components [2,4,12,18]. These tests were developed (i.e., CRF, agility, and speed tests) or selected (i.e., muscular power and flexibility tests) to provide an inexpensive, easy-to-administer, and time-efficient battery that can be implemented by primary school teachers in routine school settings.

**Cardiorespiratory fitness.** CRF was assessed with the 6-Min Shuttle Run-Walk Test (6-MSRWT) [4,18,29]. Children needed to go back and forth between 2 cones located 20 m apart. To recall the intermittent nature of children’s habitual PA [9], children were instructed to run (back) and walk (forth) as fast as possible during 6 min. The investigators continuously verified that the children complied with this instruction. The 6 min duration was selected because similar duration of walk/run test have been used to assess CRF in youth [8]. The distance covered by the children was recorded (in m).

**Agility.** Agility was measured with the hopscotch test [4,18,29]. This test was developed to facilitate implementation in schools, as hopscotch is a familiar playground activity and many French and Belgian schools already have hopscotch markings available. The children stood with both feet in the “earth” half-circle (Supplementary Material, Figure S1). They were then instructed to put one foot (the one wanted) inside each square (45 cm per side) as fast as possible while minimizing errors (i.e., placing both feet in a single square, skipping a square, or placing only one foot in the half-circle). The test began when the children left one foot off the “earth” half-circle. When arriving in the “sky” half-circle, children needed to turn back as fast as they could and go back to the “earth” half-circle. The test ended when the children put both feet in the “earth” half-circle. The time to complete the hopscotch was recorded by the investigator, as well as the number of errors made. Children performed the test twice and only the best performance was recorded.

**Muscular power.** The Standing Broad Jump (SBJ) was used to measure muscular power of lower limbs [4,18,29,30]. Standing in the starting position immediately behind a line with feet approximately shoulder-width apart, children were prompted to jump as far as possible, and to maintain balance upon landing. No specific instructions regarding jump technique were provided. Investigators measured the distance (in cm) between the line and the nearest heel to the line at landing.

**Speed.** Speed was assessed by running as fast as possible for 5 s and measuring the distance covered [4,12,18]. The test began when the children lifted one foot off the ground. It ended 5 s after using a timer. Cones were placed on the ground every meter to record the distance covered. An investigator recorded the last cone reached as the timer sounded. Then, the distance covered (in m) was recorded. This speed test was developed to reduce the influence of premature deceleration that may occur during fixed-distance speed tests in children [31].

**Flexibility.** A simplified version of the fingertip-to-floor test was used to measure flexibility [4,12,22]. Children stood in a comfortable position, legs straight, feet together, at a hip-parallel distance. They were then instructed to progressively flex their trunk and reach down as far as possible. Children needed to maintain the final position for 3 s. Investigators continuously verified that the children’s legs were straight during the test. Results of this test were scored as follows: 5 for placing the palm of both hands on the ground; 4 for fingertips of each hand touching the ground; 3 for fingers held on the ankle (on the talus); 2 for fingertips at the middle of the tibia (i.e., between the talus and the patella); and 1 for fingers reaching the patella [4,12]. The scoring procedure was adapted compared to the original version of the fingertip-to-floor test to simplify administration.

### 2.4. Battery of Reference Tests

Five REF tests were performed to examine the concurrent and construct validity of the DIAG [13,30]. These tests were selected because they are widely recognized for assessing specific components of PF in children [2,3,13,32,33,34,35,36,37].

**Cardiorespiratory fitness.** The reduced Cooper test was performed as it is commonly preferred in schools because it is easy to administer, requires few equipment, is accessible for younger children, and carries a low risk of frustration due to early dropout [8,33,34,37]. Children were instructed to achieve the longest distance possible in 6 min, by running or walking around a team sport field [37]. The total distance covered (in m) by the children was collected.

**Agility.** Agility was measured with the quadrant jump test as it focuses on coordination, rhythm, and quick foot movements [38,39]. A quadrant was delineated on the floor, formed by 2 perpendicular lines 90 cm long. A line also indicated the starting position. Each square was numbered clockwise from 1 to 4. Children were instructed to perform successive jumps into square 1, 2, 3, and 4. The sequence was repeated, aiming to attain maximal number of repetitions within 10 s. The score obtained for the test was defined as the number of squares covered. A penalty of 0.5 point was deducted each time a quadrant line was touched. Children performed the test twice and only the best performance was recorded.

**Muscular power.** Muscular power was assessed with the original version of the SBJ [13,32,40,41]. Unlike the SBJ included in the DIAG battery, children were instructed to flex knees, swing arms backwards and then jump as far as they could, accompanying the jump with a forward swing of the arms [41]. Then, the distance (in cm) between the line and the nearest heel to the line at landing was recorded.

**Speed.** Speed was assessed with the 20 m sprint [33,34,37]. Starting in a standing position, feet behind the starting line, children were instructed to run as fast as they could until the finish line (placed 20 m beyond the starting line). The test began when the child lifted one foot off the ground and ended upon crossing the finish line. The time (in s) to cover the 20 m was recorded.

**Flexibility.** The Sit-and-Reach (SR) test was used to assess flexibility [2,3,35,36,42]. Children sat on the floor, knees and arms extended, legs touching, soles of the feet against the edge of a reach box, hands parallel to the floor and placed on top of each other with the fingertips aligned. They were then instructed to go forward as far as possible and maintain the position for at least 3 s. Then, the distance (in cm) reached on the box by the fingertips was retained, using 15 cm at the level of the feet. If the participant was unable to touch the front edge of the box with their fingertips, where the “0” mark is located, the distance was recorded as negative [42].

### 2.5. Statistical Analysis

Concurrent validity of the DIAG tests was evaluated by examining the correlations between performances on each DIAG test and the respective REF test in the 3 age groups (i.e., 6–7, 8–9, and 10–11 years old children) separately [22,30]. Depending on the distribution of the data, controlled using a Shapiro–Wilk normality test, Pearson (*r*_p_) or Spearman (*r*_s_) correlation coefficients were calculated. Pearson and Spearman correlation coefficients were interpreted according to the following thresholds: values between 0 and 0.39 indicated a weak relationship, values from 0.40 to 0.69 reflected a moderate relationship, and values between 0.70 and 1.00 signified a strong relationship [26].

Construct validity was assessed by using a two-factor ANalysis Of VAriance (ANOVA) with age (6–7 vs. 8–9 vs. 10–11 years) and sex (girls vs. boys) as factors provided that the data followed a Gaussian distribution and the assumption of homogeneity of variances, verified with Levene’s test, was met. When a significant main effect was identified, Tukey’s *post-hoc* tests were conducted to determine pairwise differences between specific age groups. When the data did not follow a Gaussian distribution, construct validity was assessed using the non-parametric Mann–Whitney U test to evaluate the effect of sex, and the Kruskal–Wallis test to examine the effect of age. When a significant main effect of age was identified, Dwass–Steel–Critchlow–Fligner *post-hoc* tests were applied to determine pairwise differences between specific age groups. Data are presented in tables and figures as mean ± standard deviation or median [25th, 75th percentile], depending on the distribution. *p*-values below 0.05 were considered as significant. Statistical analyses were conducted with Jamovi (version 2.4.14) and graphics were created using GraphPad Prism 8.0 (GraphPad Software, San Diego, CA, USA).

## 3. Results

### 3.1. Participants Characteristics

**Figure 1:**
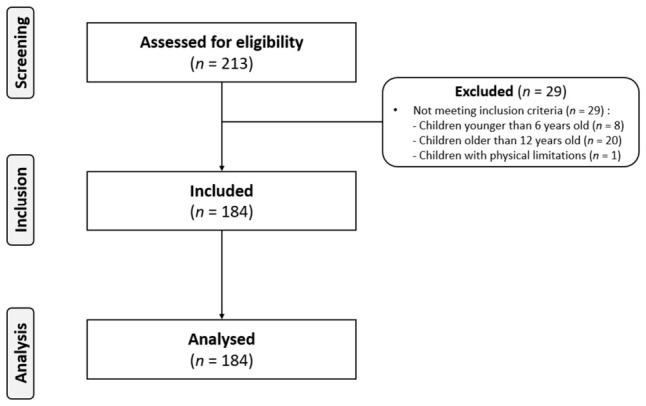
Flow diagram of participant selection in accordance with the Strengthening The Reporting of OBservational studies in Epidemiology (STROBE) guidelines [43].

The final sample included 184 children (*n* = 62 for 6–7 years, *n* = 66 for 8–9 years, and *n* = 56 for 10–11 years). The flow diagram is illustrated in Figure 1.

The socio-demographic and anthropometric characteristics of the included children are presented in Table 2.

### 3.2. Concurrent Validity

**Figure 2:**
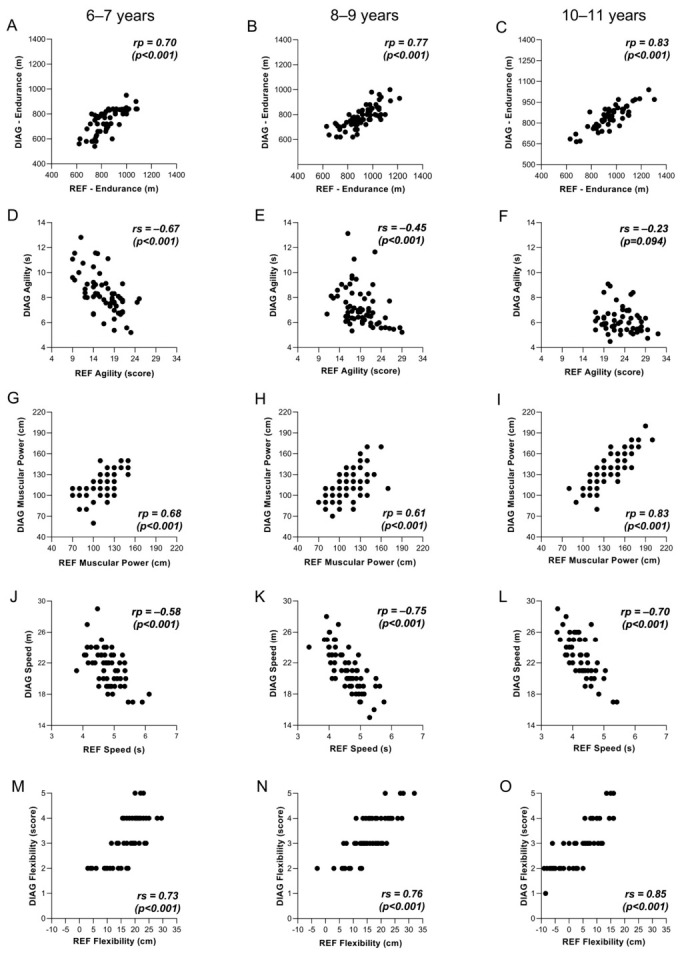
Relations between performances obtained during the Diagnoform^®^ Kid (DIAG) and reference (REF) tests in the 3 age groups (6–7, 8–9, and 10–11 years): (**A**–**C**) relationships between the cardiorespiratory fitness tests, (**D**–**F**) relationships between the agility tests, (**G**–**I**) relationships between the muscular power tests, (**J**–**L**) relationships between the speed tests, (**M**–**O**) relationships between the flexibility tests. Pearson (*r_p_*) or Spearman (*r_s_*) correlation coefficients are reported depending on the distribution of the data.

Concurrent validity was examined separately for each of the 3 age groups by correlating performance on each DIAG test with that of its corresponding REF test. Figure 2 presents individual data and Pearson (*r_p_*) or Spearman (*r_s_*) correlation coefficients for each age group for CRF, agility, muscular power, speed, and flexibility.

The correlation coefficients reveal varying levels of concurrent validity across PF components and age groups. For CRF, strong correlations were observed in all age groups between the tests of the two batteries (*r_p_* between 0.70 and 0.83; *p* < 0.001; Figure 2A–C). Agility showed moderate correlations in the younger groups (6–7 years: *r_s_* = −0.67 and 8–9 years: *r_s_* = −0.45; both *p* < 0.001), whereas in the 10–11 years group, the correlation was weak and showed only a trend toward significance (*r_s_* = −0.23; *p* = 0.094; Figure 2D–F). For muscular power, moderate correlations were found in the younger groups (6–7 years: *r_p_* = 0.68 and 8–9 years: *r_p_* = 0.61; both *p* < 0.001), while correlation was strong in the older group (*r_p_* = 0.83; *p* < 0.001; Figure 2G–I). Speed demonstrated a moderate correlation in the 6–7 years group (*r_p_* = −0.58; *p* < 0.001) and strong correlations in the older age groups (8–9 years: *r_p_* = −0.75 and 10–11 years: *r_p_* = −0.70; both *p* < 0.001; Figure 2J–L). Finally, flexibility showed strong correlations across all age ranges between the DIAG and REF tests (*r_s_* between 0.73 and 0.85; *p* < 0.001; Figure 2M–O).

### 3.3. Construct Validity—Responsiveness to Age and Sex Effects

Construct validity was evaluated by examining the responsiveness of each DIAG test and its corresponding REF test to age and sex effects using a two-factor ANOVA when the data followed a Gaussian distribution. When the distribution was non-Gaussian, the non-parametric Mann–Whitney U test and Kruskal–Wallis test were used to assess the effects of sex and age, respectively. Supplementary Material Figure S2 graphically presents the data, in girls and boys for the different age groups, from top to bottom for the CRF, agility, muscular power, speed, and flexibility tests. Table 3 presents the performances on the 5 tests of the DIAG and the REF PF batteries according to age group and sex.

For CRF, the age × sex interaction was not significant for either the DIAG test or the REF test (ANOVA, *p* = 0.554 and *p* = 0.138, respectively). In contrast, significant main effects of age and sex were observed for both tests (ANOVA, both *p* < 0.001). Regarding age, *post-hoc* analyses revealed significant differences between all age groups for the DIAG test (all *p* < 0.01), with performance increasing with age. In contrast, for the REF test, the 6–7 years group demonstrated significantly lower performance compared to the two older groups (*p* < 0.01), whereas no significant difference was found between the 8–9 and 10–11 years groups (*p* = 0.138, Table 3). Regarding sex effect, performance was higher in boys compared to girls for both batteries (*p* < 0.001).

For agility, the data did not follow a Gaussian distribution; therefore, non-parametric tests were used. A significant effect of age was observed for both the DIAG (Kruskal–Wallis, *p* < 0.001) and REF tests (*p* < 0.001). For both DIAG and REF tests, Dwass–Steel–Critchlow–Fligner *post-hoc* analyses indicated significant differences between all age groups (all *p* < 0.01), with performance increasing with age. No significant effect of sex was found for either the DIAG (hopscotch; Mann–Whitney U, *p* = 0.146) or the REF test (quadrant jump; *p* = 0.122).

For muscular power, the age × sex interaction was not significant for either the DIAG or REF tests (ANOVA, *p* = 0.991 and *p* = 0.573, respectively). In contrast, significant main effects of both age and sex were observed for both tests (ANOVA, both *p* < 0.001). *Post-hoc* analyses showed a significantly higher performance in the oldest group compared to the younger age groups (all *p* < 0.001), but no difference between the 6–7 and 8–9 years groups for either the DIAG or REF test (*p* = 0.987 and *p* = 0.515, respectively). Performance was higher in boys compared to girls for both batteries (*p* < 0.001).

For speed, the age × sex interaction was not significant for either the DIAG or the REF tests (ANOVA, *p* = 0.477 and *p* = 0.523, respectively), while significant main effects of both age and sex were observed for both tests (ANOVA, both *p* < 0.001). Regarding age, *post-hoc* analyses indicated a significant difference between all age groups for the REF test (all *p* < 0.05), with performance increasing with age. For the DIAG test, the oldest group outperformed both younger groups (both *p* < 0.05), although no significant difference was observed between the 6–7 and 8–9 years groups (*p* = 0.792). Regarding sex, performance was higher in boys compared to girls for both batteries (*p* < 0.001).

For flexibility, the data did not follow a Gaussian distribution, thus non-parametric tests were also used. A significant effect of sex was observed for both the DIAG (Mann–Whitney U, *p* = 0.019) and the REF (*p* = 0.018) tests. Given the significant effect of sex, age effects were examined separately for girls and boys. For girls, the Kruskal–Wallis test did not reveal a significant age effect for either the DIAG or the REF test (*p* = 0.721 and *p* = 0.302, respectively). In contrast, for boys, a significant age effect was observed for the REF test (*p* = 0.007) but not for the DIAG test (*p* = 0.084). For the REF test, Dwass–Steel–Critchlow–Fligner *post-hoc* analyses indicated a significantly higher performance in boys aged 6–7 than 10–11 years (*p* = 0.011), but no significant difference between boys aged 6–7 and 8–9 years or between 8–9 and 10–11 years (all *p* > 0.05).

## 4. Discussion

This cross-sectional study examined the concurrent and construct validity of the DIAG tests. Overall, the results are indicative of both concurrent and construct validity of the 5 DIAG tests, with the exception of a lower responsiveness of the simplified fingertip-to-floor test to age-related changes in boys.

For CRF, a strong level of correlation between the 6-MSRWT and the reduced Cooper test was observed in the 3 age groups (Figure 2A–C), suggesting good concurrent validity of the DIAG test. Regarding construct validity, CRF increased significantly between 6–7 years and the two older groups for both batteries, while a significant difference between 8–9 and 10–11 years was observed for the 6-MSRWT, but not for the reduced Cooper test (Table 3). This slight difference in age responsiveness could be related to the type of test [4,12,37]. More specifically, CRF tends to increase with age when shuttle-run tests are used, whereas it may stagnate for continuous tests (e.g., reduced Cooper) [4,12,37]. This difference might be partly explained by the greater anaerobic contribution required in the shuttle run tests due to the repeated deceleration, stopping, and acceleration necessary to turn around the cones every 20 m [28,45]. Anaerobic performance is indeed dependent on anaerobic enzyme activity, muscular mass, neuromuscular transmission, and contractile muscle speed, all of which increase with biological maturation during childhood and adolescence [46,47,48,49]. Beyond physiological factors, motivational aspects could also contribute to the difference observed between the CRF tests. Indeed, it is well established that motivation to engage in PA decreases around 10–11 years compared with 8–9 years, and continuous exercises may be perceived as less motivating than intermittent efforts, which are more natural and engaging for children [9,10,37,50].

For agility, significant correlations were observed between the hopscotch and hexagon tests in the younger groups (Figure 2D–F), which suggests concurrent validity of the DIAG test. However, a weak correlation was found in the 10–11-year-old group, which may be explained by a potential ceiling effect (i.e., the hopscotch test may be too easy for older children) [51]. Regarding construct validity, performance at both agility tests improved similarly with advancing age and no effect of sex was reported (Table 3).

For muscular power, regarding concurrent validity, a moderate correlation was observed in the younger groups (Figure 2G,H), while it was strong for the 10–11 years group (Figure 2I). This might be due to the difference in instructions given to the children. Indeed, children were instructed to flex the knees before jumping in the original SBJ [41], whereas in the DIAG test (SBJ without instruction), no execution instructions were given to the children, in line with the DIAG guidelines [18,52,53]. This absence of specific instructions might have allowed younger children to adopt a more natural movement pattern and to use “plyometric rebound” before jumping [54]. This plyometric contraction could explain (a) the higher performance observed for certain children in the DIAG test compared to the original version of SBJ (Figure 2G,H and Table 3), and thus (b) the moderate correlation in the younger groups. Regarding construct validity, both SBJ tests detected similarly the higher level of muscular power in the oldest group compared with the younger groups, and boys outperformed girls (Table 2).

For speed, significant levels of correlation (i.e., moderate to strong) were found in all age groups (Figure 2J–L) suggesting good concurrent validity of the DIAG test. Performance measured during sprint tests depends on several factors, including acceleration, maximum speed, and deceleration [55,56]. Acceleration is typically measured over the first 10 m, while maximum speed is generally reached between 15 and 20 m in non-specialists [57,58]. Although both speed tests similarly assess initial acceleration and maximal speed, in the REF test (i.e., the 20 m sprint), children may have begun to slow down as they approached the finish line. This tendency to decelerate could partly explain the moderate correlation observed in younger children [31]. Regarding construct validity, for both speed tests, older children were faster than younger ones, and boys outperformed girls (Table 3).

For flexibility, a strong correlation was observed in each age group between the simplified-fingertip-to-floor and the SR tests (Figure 2M–O), which indicates a good concurrent validity of the DIAG test. Regarding construct validity, although an effect of sex was observed in both flexibility tests, only the SR test detected a decrease in flexibility between 6–7 years and 10–11 years in boys. The inability of the DIAG test to detect this decrease in flexibility may be due to the lower measurement precision of this simplified version compared to the original fingertip-to-floor test [4,22]. Indeed, the result of the original fingertip-to-floor test expresses results in centimeters [22], whereas the DIAG version uses a score ranging from 0 to 5 [4,18,52]. In line with these results, we recommend that DIAG creators introduce intermediate levels between the five existing scores to improve test responsiveness.

### Strengths, Limits, and Future Directions

This cross-sectional study has both strengths and limitations. Beyond its strong ecological validity, it aligns with the STrengthening the Reporting of OBservational studies in Epidemiology (STROBE) guidelines (Supplementary Material, Table S1), which support high reporting quality and methodological rigor [43]. Furthermore, the present findings provide preliminary evidence for the concurrent and construct validity of the DIAG battery and highlight its potential to address practical challenges in school-based PF assessment. In particular, the DIAG is more time-efficient (i.e., ~60 min vs. ~120 min for some existing PF batteries for children) [13], relies only on equipment commonly available in physical education classes (e.g., cones and whistle, whereas some other PF batteries require dynamometers, audio pacing systems, or skin-fold calipers) [13], and may better align with children’s natural tendency toward intermittent exercise. Nevertheless, potential limitations should be considered when interpreting the results. Indirect methods (i.e., field tests) were used as reference measures to test the concurrent and construct validity of the DIAG battery [2,13,32,33,34,35,36,37]. These field PF tests are well-established methods to assess PF level, but they remain less accurate than direct measurements of PF (e.g., peak oxygen uptake for CRF) [8]. Regarding future perspectives, since it is important to monitor PF throughout the lifespan and in people with disabilities, future cross-sectional studies should assess the concurrent and construct validity of the Diagnoform^®^ tests batteries dedicated to different age groups (i.e., Diagnoform^®^ Tonic for adolescents, Diagnoform^®^ Actif for adults, Diagnoform^®^ Santé for older adults) and to individuals with motor disabilities (i.e., Diagnoform^®^ Handi). These batteries share the same practical advantages as the DIAG (i.e., easy administration, a large reference database, and an online platform) [53,59]. Finally, future studies could investigate the validity of the Diagnoform^®^ Kid battery in children of different BMI classes. This could help determine whether the observed associations between the DIAG and the reference tests remain consistent across different anthropometric profiles.

## 5. Conclusions

The findings of this cross-sectional study support preliminary evidence for the concurrent validity and the construct validity of the Diagnoform^®^ Kid, except for the simplified fingertip-to-floor test, whose lower responsiveness to age suggests the need to refine its scoring system. Overall, the results suggest that the Diagnoform^®^ Kid is a valid assessment tool and can be recommended for routine use by professionals to monitor PF in healthy children, given its strong association with health status. The Diagnoform^®^ Kid supports this public health mission by providing a valid and reliable method for assessing children’s physical fitness [18] that requires minimal equipment, is quick to administer, and offers age- and sex-specific reference values based on a large sample of children. Finally, it benefits from an advanced digital platform that enables professionals to efficiently compare individual or group PF levels with age- and sex-matched peers.

## Figures and Tables

**Table 1:** Inclusion and exclusion criteria.

Inclusion Criteria	Exclusion Criteria
Aged between 6.00 to 11.99 years. Free from any physical limitations preventing the performance of PF tests.	Aged <6.00 or ≥12.00 years. Condition affecting PF test performance (e.g., chronic disease, musculoskeletal disorders, developmental disorders).

PF: Physical Fitness.

**Table 2:** Sociodemographic and anthropometric characteristics of the children included in the analysis (*n* = 184).

	6–7 Years		8–9 Years		10–11 Years	
	Girls	Boys	Girls	Boys	Girls	Boys
Sample size (*n*)	31	31	34	32	29	27
Height (cm)	120 ± 5.4	123 ± 6.4	134 ± 6.7	135 ± 7.3	151 ± 6.6	146 ± 7.8
Body mass (kg)	22.9 ± 3.5	25.7 ± 4.9	32.50 ± 2.3	31.9 ± 6.6	50.1 ± 13.0	42.8 ± 10.3
BMI class (*n*, %)						
Normal range	26 (83.9%)	26 (83.9%)	20 (58.8%)	24 (75.0%)	15 (51.8%)	16 (59.3%)
Overweight	4 (12.9%)	3 (9.7%)	13 (38.2%)	7 (21.9%)	7 (24.1%)	8 (29.6%)
Obesity	1 (3.2%)	2 (6.4%)	1 (3.0%)	1 (3.1%)	7 (24.1%)	3 (11.1%)

BMI: Body Mass Index. Children were classified into BMI classes according to age- and sex-specific cut-off points [44]. The 184 children included in the analyses were recruited from four schools and a non-profit association as follows: school 1 (*n* = 32), school 2 (*n* = 42), school 3 (*n* = 26), school 4 (*n* = 30), and the association (*n* = 54). Data are presented as mean ± standard deviation for height and body mass.

**Table 3:** Performance in the Diagnoform^®^ Kid (DIAG) and the reference (REF) physical fitness battery according to age and sex.

	6–7 Years	8–9 Years	10–11 Years	Girls	Boys
**Cardiorespiratory fitness**					
DIAG–6-MSRWT (m)	738.9 ± 96.6 ^b,c^	780.1 ± 87.2 ^a,c^	846.3 ± 80.9 ^a,b^	754.1 ± 93.6	820.4 ± 91.9 ^d^
REF–Reduced Cooper (m)	847.8 ± 108.7 ^b,c^	914.5 ± 127.3 ^a^	955.4 ± 136.3 ^a^	861.3 ± 120.2	949.6 ± 127.4 ^d^
**Agility**					
DIAG–Hopscotch (s)	8.1 [7.4, 9.1] ^b,c^	6.8 [6.1, 7.9] ^a,c^	6.0 [5.4, 6.6] ^a,b^	6.7 [5.9, 8.0]	7.0 [6.2, 8.3]
REF–Quadrant jump (score)	16.8 [14.0, 19.4] ^b,c^	19.0 [16.5, 21.5] ^a,c^	23.3 [20.0, 26.0] ^a,b^	19.0 [15.5, 22.0]	20.0 [17.4, 22.8]
**Muscular power**					
DIAG–SBJ no instructions (cm)	116.5 ± 19.2 ^c^	116.8 ± 21.7 ^c^	138.8 ± 25.6 ^a,b^	115.1 ± 21.5	132.0 ± 24.1 ^d^
REF–SBJ with instructions (cm)	110.7 ± 21.6 ^c^	114.7 ± 21.2 ^c^	138.9 ± 25.6 ^a,b^	114.0 ± 24.2	127.7 ± 25.5 ^d^
**Speed**					
DIAG–5 s sprint (m)	21.4 ± 2.4 ^c^	21.1 ± 2.7 ^c^	22.7 ± 2.7 ^a,b^	21.1 ± 2.8	22.4 ± 2.5 ^d^
REF–20 m sprint (s)	4.8 ± 0.4 ^b,c^	4.6 ± 0.5 ^a,c^	4.3 ± 0.4 ^a,b^	4.7 ± 0.5	4.4 ± 0.4 ^d^
**Flexibility**					
DIAG–SFTF (score)	3.0 [2.2, 4.0]	3.0 [3.0, 4.0]	3.0 [2.0, 3.2]	3.0 [3.0, 4.0]	3.0 [2.0, 3.0] ^d^
REF–SR (cm)	18.0 [14.0, 20.9] ^e^	14.8 [11.4, 18.9]	15.3 [6.9, 18.5] ^f^	17.0 [12.5, 21.4]	15.0 [9.9, 18.5] ^d^

6-MSRWT: 6-Min Shuttle Run Walk Test; SBJ: Standing Broad Jump; SFTF: Simplified Fingertip-To-Floor, SR: Sit-and-Reach; ^a^ significantly different from 6–7 years (*p* < 0.05); ^b^ significantly different from 8–9 years (*p* < 0.05); ^c^ significantly different from 10–11 years (*p* < 0.05); ^d^ significantly different from girls (*p* < 0.05); ^e^ significantly different from 10–11 years in boys (*p* < 0.05); ^f^ significantly different from 6–7 years in boys (*p* < 0.05). Data are presented as mean ± standard deviation or median [25th, 75th percentile], depending on the distribution.

## Data Availability

The raw data supporting the conclusions of this article will be made available by the authors upon request.

## Supplementary Materials

The following supporting information can be downloaded at: https://www.mdpi.com/article/10.3390/jfmk11030256/s1, Table S1. STROBE Statement—checklist of items that should be included in reports of observational studies; Figure S1. Representation of the hopscotch test of the Diagnoform^®^ Kid; Figure S2. Results from the Diagnoform^®^ Kid (DIAG) and the reference (REF) tests according to age and sex.

## Abbreviations

The following abbreviations are used in this manuscript:

6-MSRT	6-Min Shuttle Run-Walk Test
ACSM	American College of Sports Medicine
ANOVA	ANalysis Of VAriance
BMI	Body Mass Index
CRF	CardioRespiratory Fitness
DIAG	DIAGnoform^®^ Kid
PA	Physical Activity
PF	Physical Fitness
REF	REFerence
SBJ	Standing Broad Jump
SFTF	Simplified Fingertip-To-Floor
SR	Sit-and-Reach
STROBE	Strengthening the Reporting of OBservational studies in Epidemiology
