# Supplementary material for: Concurrent and Construct Validity of the Diagnoform® Kid Physical Fitness Test Battery in Primary School Children
Source: J Funct Morphol Kinesiol. 2026 Jun 27;11(3):256. doi: 10.3390/jfmk11030256 (PMC13398135; doi:10.3390/jfmk11030256)
Supplement: Supplementary file 1 [file jfmk-11-00256-s001.zip › jfmk-4387344-supplementary.pdf]

Table S1. STROBE Statement—checklist of items that should be included in reports of observational studies

	Item No.	Recommendation	Page No.	Relevant text from manuscript
**Title and abstract**	1	(*a*) Indicate the study's design with a commonly used term in the title or the abstract	1	Line 16: *"this cross-sectional study"*
		(*b*) Provide in the abstract an informative and balanced summary of what was done and what was found	1	Lines 14-32
**Introduction**				
Background/rationale	2	Explain the scientific background and rationale for the investigation being reported	1-2	Lines 36-85
Objectives	3	State specific objectives, including any prespecified hypotheses	2	Lines 86-90
**Methods**				
Study design	4	Present key elements of study design early in the paper	2<br>3	Line 86: "*this cross-sectional study*"<br>Lines 89-108
Setting	5	Describe the setting, locations, and relevant dates, including periods of recruitment, exposure, follow-up, and data collection	3	Lines 93-98
Participants	6	(*a*) *Cohort study*—Give the eligibility criteria, and the sources and methods of selection of participants. Describe methods of follow-up<br>*Case-control study*—Give the eligibility criteria, and the sources and methods of case ascertainment and control selection. Give the rationale for the choice of cases and controls<br>*Cross-sectional study*—Give the eligibility criteria, and the sources and methods of selection of participants	3	Table 1 (Page 3)
		(*b*) *Cohort study*—For matched studies, give matching criteria and number of exposed and unexposed<br>*Case-control study*—For matched studies, give matching criteria and the number of controls per case	N.A.	N.A.

Variables	7	Clearly define all outcomes, exposures, predictors, potential confounders, and effect modifiers. Give diagnostic criteria, if applicable	3-5	Line 124 (Page 3) – Line 220 (Page 5)
Data sources/ measurement	8*	For each variable of interest, give sources of data and details of methods of assessment (measurement). Describe comparability of assessment methods if there is more than one group	3-5	Line 124 (Page 3) – Line 220 (Page 5)
Bias	9	Describe any efforts to address potential sources of bias	3	Lines 124-131
Study size	10	Explain how the study size was arrived at	3<br>6	Lines 108-117<br>Figure 1
Quantitative variables	11	Explain how quantitative variables were handled in the analyses. If applicable, describe which groupings were chosen and why	3<br>5-6	Lines 108-110<br>Lines 221-243
Statistical methods	12	(*a*) Describe all statistical methods, including those used to control for confounding	5-6	Lines 221-243
		(*b*) Describe any methods used to examine subgroups and interactions	5-6	Lines 221-243
		(*c*) Explain how missing data were addressed	6	**Figure 1**
		(*d*) *Cohort study*—If applicable, explain how loss to follow-up was addressed<br>*Case-control study*—If applicable, explain how matching of cases and controls was addressed<br>*Cross-sectional study*—If applicable, describe analytical methods taking account of sampling strategy	N.A.	N.A.
		(*e*) Describe any sensitivity analyses	6	Lines 230-243
**Results**				
Participants	13*	(a) Report numbers of individuals at each stage of study—eg numbers potentially eligible, examined for eligibility, confirmed eligible, included in the study, completing follow-up, and analysed	6<br>6	Lines 246-247<br>**Figure 1**
		(b) Give reasons for non-participation at each stage	6	**Figure 1**
		(c) Consider use of a flow diagram	6	**Figure 1**
Descriptive data	14*	(a) Give characteristics of study participants (eg demographic, clinical, social) and information on exposures and potential confounders	6	**Table 2**

		(b) Indicate number of participants with missing data for each variable of interest	N.A.	N.A.
		(c) *Cohort study*—Summarise follow-up time (eg, average and total amount)	N.A.	N.A.
Outcome data	15*	*Cohort study*—Report numbers of outcome events or summary measures over time	N.A.	N.A.
		Case-control study—Report numbers in each exposure category, or summary measures of exposure	N.A.	N.A.
		Cross-sectional study—Report numbers of outcome events or summary measures	6	**Figure 1**
Main results	16	(*a*) Give unadjusted estimates and, if applicable, confounder-adjusted estimates and their precision (eg, 95% confidence interval). Make clear which confounders were adjusted for and why they were included	7-10	Line 261 – Line 326
		(*b*) Report category boundaries when continuous variables were categorized	N.A.	N.A.
		(*c*) If relevant, consider translating estimates of relative risk into absolute risk for a meaningful time period	N.A.	N.A.

*Give information separately for cases and controls in case-control studies and, if applicable, for exposed and unexposed groups in cohort and cross-sectional studies.

Note: N.A. Not Applicable. An Explanation and Elaboration article discusses each checklist item and gives methodological background and published examples of transparent reporting. The STROBE checklist is best used in conjunction with this article (freely available on the Web sites of PLoS Medicine at http://www.plosmedicine.org/, Annals of Internal Medicine at http://www.annals.org/, and Epidemiology at http://www.epidem.com/). Information on the STROBE Initiative is available at www.strobe-statement.org.

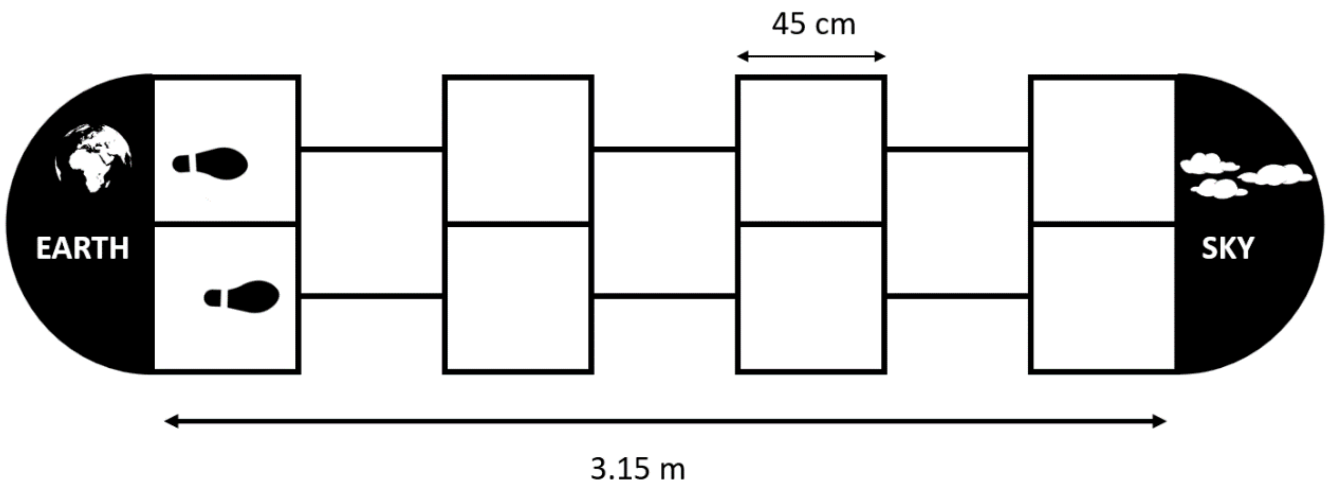

Figure S1. Representation of the hopscotch test of the Diagnoform® Kid.

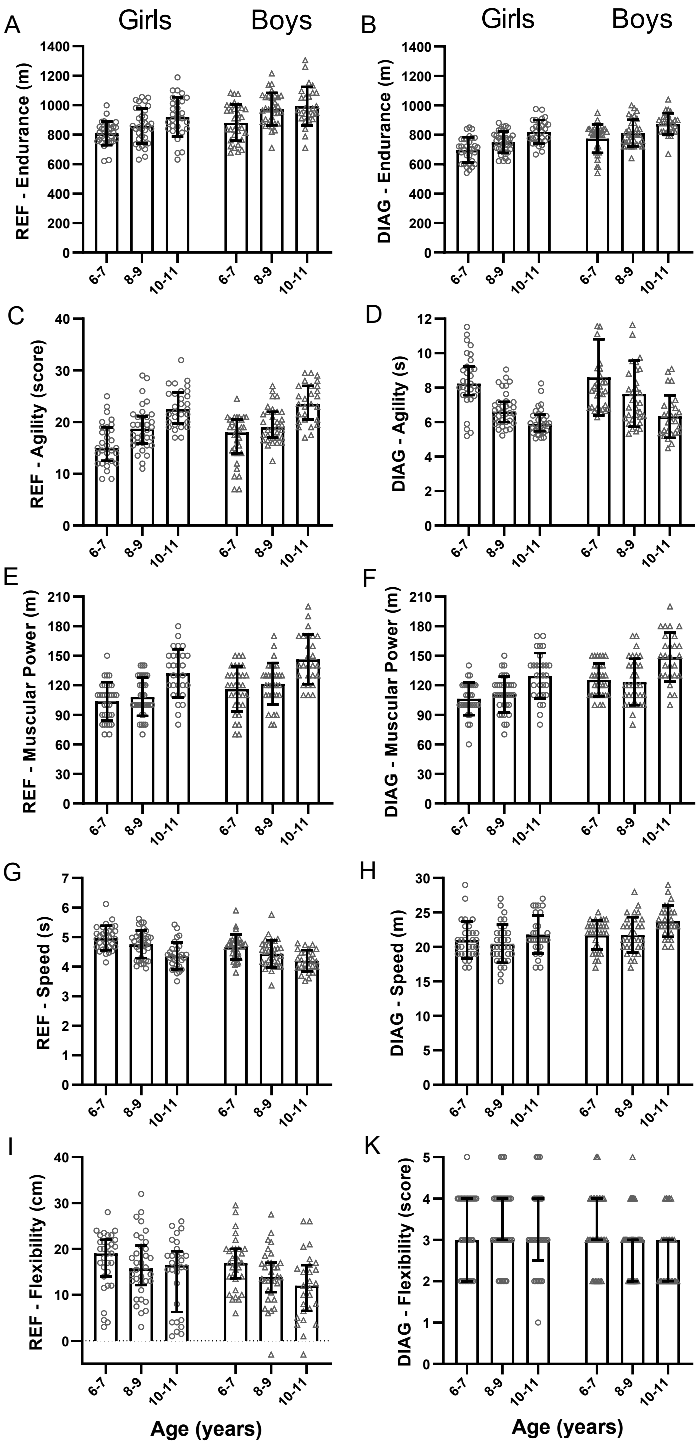

Figure S2. Results from the Diagnoform® and the reference tests according to age and sex.

Note: DIAG: DIAGnoform®, REF: REFerence. Data are presented as mean ± standard deviation or median [25th, 75th percentile], depending on the distribution. Results from the reference tests are shown in the left panels and those from the Diagnoform® tests in the right panels. (**A–B**) mean ± standard deviation of the cardiorespiratory fitness tests, (**C–D**) median [25th, 75th percentile] of the agility tests, (**E–F**) mean ± standard deviation of the muscular power tests, (**G–H**) mean ± standard deviation of the muscular speed tests, (**I–K**) median [25th, 75th percentile] of the flexibility tests.
